# Acquired Genotype‐Positive Long QT Syndrome After Pediatric Heart Transplantation

**DOI:** 10.1111/petr.70075

**Published:** 2025-04-02

**Authors:** Nicholas V. Barresi, Jessica Sebastian, Gaurav Arora, Brian Feingold

**Affiliations:** ^1^ Division of Pediatric Cardiology Children's Hospital of Pittsburgh of UPMC Pittsburgh Pennsylvania USA; ^2^ Division of Genetics and Genomic Medicine Children's Hospital of Pittsburgh of UPMC Pittsburgh Pennsylvania USA

**Keywords:** channelopathy, congenital long QT syndrome, pediatric heart transplantation

## Abstract

**Background:**

Congenital long QT syndrome (LQTS) is rare but significant, as it carries a risk for ventricular arrhythmias and sudden cardiac death. Its diagnosis can be made clinically by serial ECGs, ambulatory ECG monitoring, and exercise stress testing; however, genetic testing is confirmatory in the majority of cases.

**Methods:**

Here, we describe a rare case of phenotype‐positive LQTS in a 6‐year‐old heart transplant recipient, confirmed 5 years after transplantation to be genotype‐positive and thus “acquired” from the transplanted heart.

**Results:**

Recognition of a persistently prolonged QTc interval on the recipient's serial ECGs led to ambulatory ECG monitoring and exercise stress testing—both of which were suspicious for LQTS. Ultimately, genetic evaluation and cardiac biopsy were obtained and resulted positive for a *KCNQ1* pathogenic variant associated with Type 1 LQTS.

**Conclusion:**

Recognition of persistent, otherwise unexplained, ECG abnormalities can prompt genetic analysis of the allograft, leading to the potential life‐saving diagnosis of a channelopathy.

**Abbreviations**
AED: automated external defibrillator
AVSD: atrioventricular septal defect
CAV: cardiac allograft vasculopathy
LQT1: Type 1 long QT syndrome
LQTS: Long QT Syndrome
PLN: phospholambin

## 1 Introduction

A long QTc interval on ECG is frequently encountered and the etiology is usually acquired, most often secondary to medications or electrolyte abnormalities [1]. Congenital (genotype‐positive, phenotype‐positive) long QT syndrome (LQTS), on the other hand, is an inherent disorder of ventricular myocardial repolarization that can lead to ventricular arrhythmias and an increased risk of sudden cardiac death [2]. This is much rarer with a prevalence of 1 in 2000 live births [3]. Pathogenic variants in at least 17 genes have been identified in congenital LQTS [4]. Of these, mutations in the KCNQ1 gene are most common, leading to malfunction of potassium ion channels in the myocardium and a diagnosis of Type 1 Long QT Syndrome (LQT1) [5].

In this report, we present a case of congenital (genotype‐positive, phenotype‐positive) LQTS acquired after heart transplantation by a 6‐year‐old recipient. The diagnosis was made 5 years after heart transplantation when continued observation of a prolonged QTc interval on ECG prompted genetic testing for LQTS utilizing donor cardiac tissue. To our knowledge, this is the first reported case of “acquired” genotype‐positive, phenotype‐positive LQTS after heart transplantation.

## 2 Case Report

A 6‐year‐old child with a history of partial atrioventricular septal defect (AVSD) with parachute mitral valve underwent orthotopic heart transplantation. The child had previously undergone repair of the AVSD, which was complicated by complete AV block necessitating pacemaker placement, as well as progressive mitral valve stenosis and insufficiency requiring mechanical mitral valve replacement. The child then developed severe left ventricular dysfunction and heart failure, prompting transplantation. The posttransplant course was largely uncomplicated, with only a single episode of pneumonia and no acute rejection or posttransplant cardiac allograft vasculopathy (CAV) while on maintenance immunosuppression of tacrolimus and sirolimus.

**FIGURE 1:**
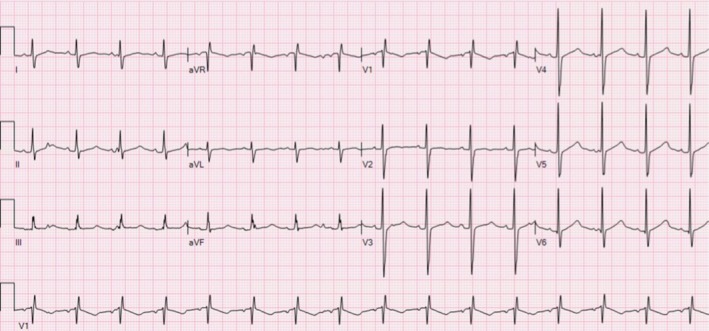
ECG of recipient on post‐op day 0 following orthotopic heart transplant. Re‐measured and calculated QTc was 506 ms. Standard calibration of ECG including paper speed of 25 mm/s, voltage of 10 mm/mV, and low‐pass filter setting of 150 Hz. QT prolongation in the immediate post‐op setting is often non‐specific for the same reasons as it is in the donor—which are common to the ICU setting.

At a follow‐up clinic visit 5 years after heart transplant, the patient was noted to have an elevated QTc interval on ECG. Upon further review, it was discovered that the first ECG posttransplant on post‐op day 0 showed a QTc of 506 ms (Figure 1), and all ECGs thereafter consistently demonstrated a prolonged QTc between 470 and 490 ms (confirmed by recalculation by a single reviewer utilizing lead II as to eliminate inter‐observer differences in calculation). This discovery prompted ambulatory ECG monitoring, which revealed automated QTc calculations that were prolonged with an average QTc of 490 ms and 98.8% of QTc > 450 ms. The only QTc prolonging medication that our patient was taking was tacrolimus, with 12‐h target levels of 2–4 ng/mL. After consultation with our electrophysiology team and discussion with the patient's family, genetic testing of the donor heart was sent using two endomyocardial biopsy specimens obtained during surveillance cardiac catheterization. This was positive for a *KCNQ1* pathogenic variant (c.1552 C>T,p.R518*) that is associated with LQT1. Genetic testing for this variant was also completed on the recipient's blood to rule out any potential recipient contribution to the genetic analysis. This testing was negative. The recipient has no family history of Long QT Syndrome or congenital deafness.

**FIGURE 2:**
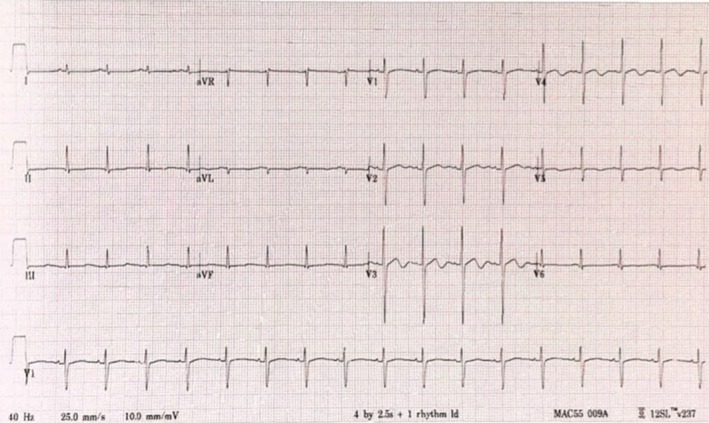
ECG of the donor heart. The re‐calculated QTc measured 510 ms, though the non‐specific T‐wave flattening in the inferolateral leads makes its exact measurement challenging.

Review of the donor data available at the time of transplantation revealed that the donor was a school‐aged child who suffered brain death after being hit by a car. The available donor medical history indicated that the child was previously healthy with no reported history of syncope. An ECG from the donor's hospitalization was available for review and showed a prolonged QTc interval of 510 ms (Figure 2).

To date, our patient has had no clinical, sentinel long QT events. They were started on nadolol to decrease ventricular arrhythmia risk, but this was discontinued due to persistent fatigue. A Bruce treadmill stress test off beta blockade was notable for baseline QTc of 464 ms with lengthening post‐exercise consistent with LQT1 [6]. No arrhythmias or concerning symptoms with exercise were noted. The recipient has preferred to not participate in competitive sports, even before suspicion or diagnosis of LQTS. The family has been counseled on avoidance of QT prolonging medications.

## 3 Discussion

In this unique case, we have documented the transmission of congenital Long QT Syndrome due to a pathogenic variant in *KCNQ1*, from a pediatric heart donor to recipient. The p.R518* pathogenic variant in *KCNQ1* identified in this case causes QTc prolongation by severely disrupting KCNQ1 channel trafficking and function in myocytes, resulting in impaired potassium regulation of the action potential [7]. To our knowledge, transmission of congenital Long QT Syndrome with heart transplantation has not been previously reported. However, there are reports of transmission of other genetic diagnoses following transplantation, including after bone marrow and stem cell transplantation [8, 9] and liver transplantation [10]. We are aware of only one other report of an “acquired,” genetic channelopathy after heart transplantation [11] in which Brugada syndrome was diagnosed in an adult recipient whose donor suffered brain death in an unexplained car accident. In that case, a type 1 Brugada pattern was seen on posttransplant ECG, representing a departure from prior baseline. This similarly led to genetic analysis on cardiac biopsy tissue confirming the diagnosis. Additionally, there is another case of an acquired genetic mutation after heart transplant that parallels our case. In that case, a 16‐year‐old female heart transplant recipient with DiGeorge syndrome developed frequent ventricular ectopy that was not explained by rejection or graft dysfunction [12]. Considering sudden and unexplained death in the donor, genetic testing using donor DNA was conducted, revealing a likely pathogenic variant in phospholambin (PLN), associated with phospholamban cardiomyopathy in which abnormal calcium homeostasis can manifest as sudden death. While an implantable cardioverter defibrillator (ICD) was placed in that recipient for presumed secondary prevention, we do not believe the donor in our case died secondary to tachyarrhythmia and, therefore, feel that ICD placement is not currently indicated. However, the principle of utilizing genetic analysis in transplant recipients in the face of persistent clinical suspicion is a common thread to several of these cases.

QTc prolongation is commonly seen after brain death [13], likely explained by changes in autonomic nervous system influence on the heart [14], common electrolyte and metabolic disturbances after brain death, and potential hypothermia and exposure to neurosedative and anti‐epileptic medications such as dexmedetomidine, propofol, or levetiracetam [15]. Thus, it is not surprising that the donor ECG available at the time of organ allocation had a prolonged QTc interval. While the donor ECG should be considered in the context of heart organ offers, it is only one of many variables that are considered in the decision‐making process of accepting a donor heart for transplantation [16], where decision‐making occurs under time constraints and in the context of a relative scarcity of organ offers and high recipient waitlist mortality [17].

Where our case has particular importance, therefore, is not in the decision on acceptance for transplant, but rather in the follow‐up care after transplantation where we observed a persistently prolonged QTc interval. Commonly used medications in transplant recipients associated with QT prolongation include tacrolimus and sulfamethoxazole‐trimethoprim [15]. Of note, other commonly used medications like mycophenolate mofetil, sirolimus, azathioprine, prednisone, enoxaparin, rivaroxaban, coumadin, valganciclovir, amlodipine, losartan, and statins have not been classified as having a known risk for QT prolongation or Torsades de Pointes [15]. Our patient was taking tacrolimus; however, the target trough level was low at 2–4 ng/mL and the long QTc persisted. Therefore, further diagnostic evaluation was pursued. Ambulatory ECG monitoring showed automated QTc calculations that were significantly prolonged in mean QTc as well as in percent QTc intervals > 450 ms—both of which have been directly correlated with increased likelihood of congenital LQTS [18]. The exercise stress test not only showed QTc prolongation at rest but also paradoxical prolongation of the QT interval during exercise and especially in the recovery phase, which is a hallmark of LQT1 [6].

Establishing the diagnosis of LQT1 in our patient has significant medical implications due to its associated risk of syncope, aborted cardiac arrest, and sudden cardiac death. Individuals with a confirmed diagnosis of LQTS have an estimated lifetime risk of sudden cardiac death of 6%–8% [19]. Therefore, a confirmatory diagnosis of LQTS directly impacts medical management and counseling. Although our patient is not an athlete, genotype‐positive/phenotype‐positive LQTS does not necessarily preclude participation in competitive sports, according to recently published guidelines [20]. With shared decision‐making and emergency action planning with access to an automated external defibrillator (AED), competitive sports can still be an option for our patient. In some higher‐risk competitive athletes, or for those who require therapy escalation, ICD placement or adjunctive therapies with left cardiac sympathetic denervation may be considered (particularly in patients with beta‐blocker intolerance, like our patient) [21, 22]. As a transplant recipient, it is possible that our patient already had sympathetic denervation and is potentially somewhat protected from a sentinel event.

Clinically, our patient continues to do well without rejection. While it has been shown in adult heart transplant recipients that donor QT_c_ > 500 ms is independently associated with CAV at 1 year after heart transplantation [23], our patient has not shown CAV by selective coronary angiography assessed at 1, 3, and 6 years after heart transplant.

Finally, our diagnosis also has the potential impact on the *donor's* family. After diagnosis, our institution notified the procuring organ procurement organization of the diagnosis, advising them to relay this information and recommendation for evaluation of first‐degree relatives to the donor's family.

## 4 Conclusion

If a recurrent ECG abnormality persists on serial follow‐up of a cardiac transplant recipient, consideration should be made for pursuing additional testing such as ambulatory ECG monitoring, stress testing, and even genetic evaluation and biopsy, if indicated. To our knowledge, this is the first reported case of genotype‐positive Long QT Syndrome acquired by heart transplantation. This highlights the importance of carrying clinical suspicion when presented with persistently abnormal ECG post heart transplantation.

## Data Availability

The authors have nothing to report.
